# Multi-body simulation of a canine hind limb: model development, experimental validation and calculation of ground reaction forces

**DOI:** 10.1186/1475-925X-8-36

**Published:** 2009-11-23

**Authors:** Gabriele Helms, Bernd-Arno Behrens, Martin Stolorz, Patrick Wefstaedt, Ingo Nolte

**Affiliations:** 1 Institute of Metal Forming and Metal-Forming Machines (IFUM), Leibniz Universität Hannover, An der Universität 2, 30823 Garbsen, Germany; 2 Small Animal Clinic, University of Veterinary Medicine Hanover, Bischofsholer Damm 15, 30173 Hannover, Germany

## Abstract

**Background:**

Among other causes the long-term result of hip prostheses in dogs is determined by aseptic loosening. A prevention of prosthesis complications can be achieved by an optimization of the tribological system which finally results in improved implant duration. In this context a computerized model for the calculation of hip joint loadings during different motions would be of benefit. In a first step in the development of such an inverse dynamic multi-body simulation (MBS-) model we here present the setup of a canine hind limb model applicable for the calculation of ground reaction forces.

**Methods:**

The anatomical geometries of the MBS-model have been established using computer tomography- (CT-) and magnetic resonance imaging- (MRI-) data. The CT-data were collected from the pelvis, femora, tibiae and pads of a mixed-breed adult dog. Geometric information about 22 muscles of the pelvic extremity of 4 mixed-breed adult dogs was determined using MRI. Kinematic and kinetic data obtained by motion analysis of a clinically healthy dog during a gait cycle (1 m/s) on an instrumented treadmill were used to drive the model in the multi-body simulation.

**Results and Discussion:**

As a result the vertical ground reaction forces (z-direction) calculated by the MBS-system show a maximum deviation of 1.75%BW for the left and 4.65%BW for the right hind limb from the treadmill measurements. The calculated peak ground reaction forces in z- and y-direction were found to be comparable to the treadmill measurements, whereas the curve characteristics of the forces in y-direction were not in complete alignment.

**Conclusion:**

In conclusion, it could be demonstrated that the developed MBS-model is suitable for simulating ground reaction forces of dogs during walking. In forthcoming investigations the model will be developed further for the calculation of forces and moments acting on the hip joint during different movements, which can be of help in context with the *in silico *development and testing of hip prostheses.

## Background

Total hip replacement has become a common form of treatment in dogs. Depending on the implanted hip prosthesis complication rates of up to 31.7% occur [1]. Aseptic loosening of the prosthesis still poses a problem in artificial joint implants. Prosthesis loosening may cause severe pain to the patient and can induce costly intensive revision surgery. Aseptic loosening can result from abrasion particles originated from the prosthetic surface area of tribological pairings and may lead to particle-induced osteolysis [2-5]. Furthermore an inappropriate positioning of the prostheses components in femur and acetabulum can result in implant loosening. In this context unphysiological load distribution in the periprosthetic osseous regions as well as stress shielding by the prosthesis can result in bone remodeling processes [6]. In particular bone resorption is followed by an aseptic loosening of the prosthesis [7].

Improving the tribological system aims at preventing aseptic loosening, thereby increasing the implant durability [8]. The knowledge of the physiological load distribution in the canine hip joint is essential for the optimization of the tribological pairing. In this context it would be of great benefit to use computerized models like multi-body simulations (MBS) for the determination of hip joint forces and moments during different movements of a dog. This approach has the advantage of calculating forces without the necessity of invasive *in vivo *experiments for different motions. In combination with finite element analysis [9,10] the MBS can help to identify areas of high loadings in the hip joint which can be further used for an optimization of the tribological pairing of prostheses [11-13]. Additionally the MBS can provide knowledge about an optimal positioning of prosthesis components according to an optimal load distribution in the periprosthetic bone. For the realization of an MBS approach to occurring forces in the canine hip joint during the canine gait it is necessary to generate a simulation model which is validated by measured data. So far no literature data exist for the forces occurring during gait in the canine hip joint in combination with recorded kinematic data. Besides that, a direct measurement of hip joint forces is not possible without complex animal experiments [14]. Therefore we aim to validate the calculated ground reaction forces of our MBS model by means of measured ground reaction forces.

Force plate analyses have been used in many studies on humans and dogs to measure ground reaction forces and motion sequences [14-17]. In dogs, gait analyses have been conducted comparing normal and abnormal locomotion and characterizing movements in general [18,19]. Although force plate analyses allow the determination of ground reaction forces, they do not deliver direct measurements of hip joint forces.

To our knowledge no MBS for the calculation of ground reaction forces as well as forces and moments in the canine hip joints during different movements exists. For that reason, the overall goal of our research is to establish an MBS-model capable of estimating the forces acting on the canine hip joint. As a first important step we present a computerized model of the hind limbs for the simulation of ground reaction forces during a gait cycle. Therefore a three-dimensional multi-body simulation model of the canine hind limb was generated by integrating osseous components and an analogous anatomic model composed of 22 muscles. To drive the static MBS-model, kinematic data obtained by the gait analysis of a clinically healthy dog were implemented into the simulation. Data resulting from ground reaction forces during walking were obtained from force plate measurements and were used to validate the calculated ground reaction forces of the MBS-model. The simulation of ground reaction forces and its validation by force plate analysis is crucial for the forthcoming realistic calculation of forces and moments acting on the canine hip joint.

## Methods

### Setup of the multi-body simulation model of the canine hind limb

Overall, 5 dog cadavers, which had to be euthanized in the small animal clinic of the university of veterinary medicine in Hannover as a result of various non-orthopedic diseases, were used for the compilation of the multi-body simulation model.

The MBS-model was generated using the commercial software BRG.LifeMOD 2008.0.0 (Biomechanics Research Group, Inc., USA). BRG.LifeMOD is based on the software MSC.ADAMS^® ^(Mechanical Dynamics Inc., USA) and was mainly designed for human multi-body simulations [20]; nevertheless, the software can also be adapted for non-human multi-body simulations. Kinematic and kinetic data obtained by gait analysis of a clinically sound dog during a gait cycle on an instrumented treadmill (belt speed: 1 m/s) were used to drive the model in the multi-body simulation and to validate the simulated results.

### Measurement of kinematic and kinetic data

The gait analyses of the dog were carried out at the gait laboratory of the clinic for small animals in accordance with the German law governing the care and use of animals (TSchG, §8(7)).

In order to realize a physiological motion sequence of the dog in the MBS-model, the instantaneous relative positions of the bone segments as well as the ground reaction forces were captured using 3D motion detection systems. The setup consists of a 4-infrared-camera system (Vicon MX-3+, Oxford Metrics, UK) and an instrumented treadmill with four integrated 3D-force plates (Bertec CTM4-B07, Columbus-OH, USA). Vicon Nexus^® ^(Oxford Metrics, UK) software was used to record both, kinematic measurements and ground reaction forces.

**Figure 1:**
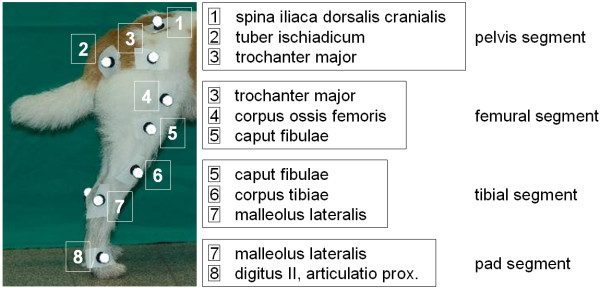
**Retroreflective markers positioned at selected bony landmarks (labels within illustration) of the hind limb**. 16 retroreflective markers (diameter: 16 mm, 8 per side) were positioned at the bony landmarks of the hind limb with double-sided adhesive tape.

For the movement functions during gait a healthy mixed-breed male dog (age: 8 years; body condition score: 5; BW: 28 kg) was labeled with 16 retroreflective markers (diameter: 16 mm, 8 per side) positioned at the bony landmarks of the hind limb with double-sided adhesive tape as shown in Figure 1. Motion capture of the dog during gait on the treadmill was carried out after calibration of the system (mean measurement error for all four cameras: 0.07 mm) at a belt speed of 1 m/s. At this speed the dog showed a steady-going, undisturbed gait. To achieve enough repeatable motion data for implementation into the MBS-system, overall 6 trials of 30 sec. duration were recorded.

**Figure 2:**
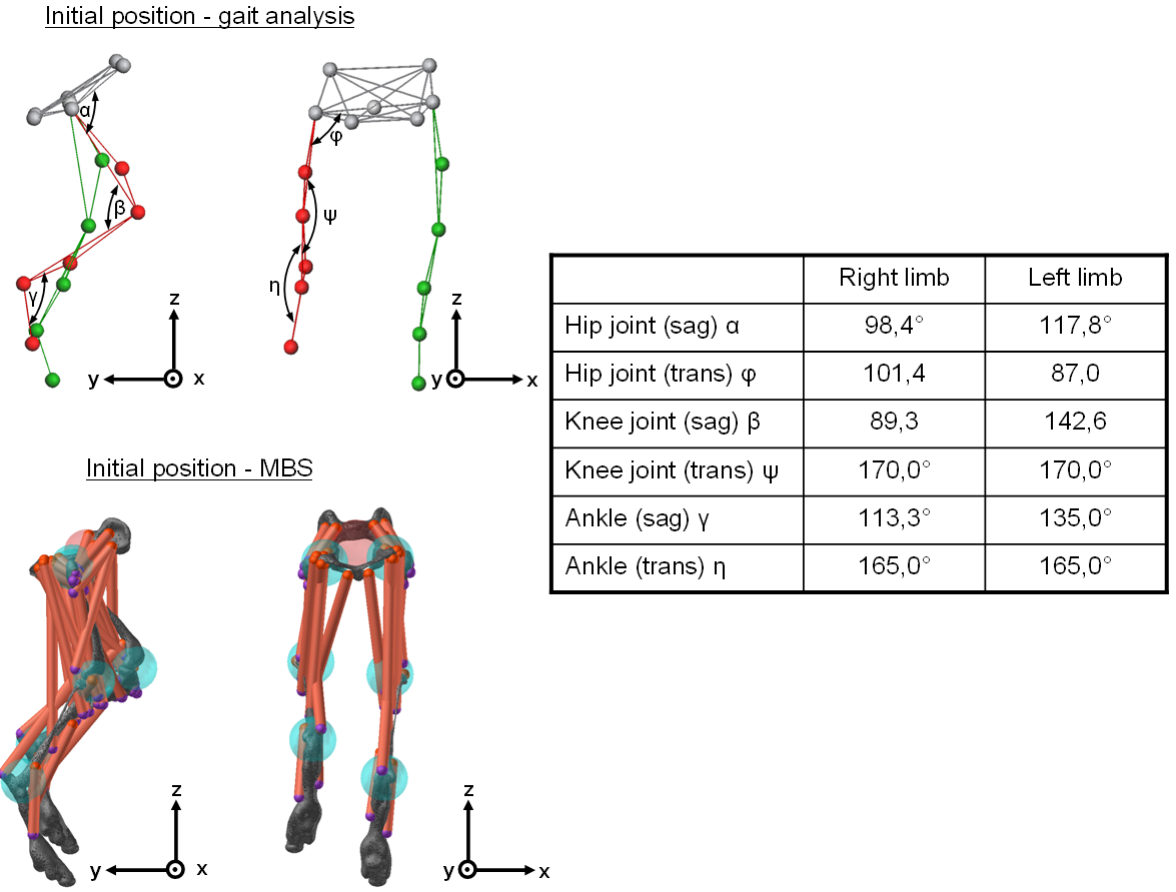
**Initial position of the canine hind limb in the gait analysis and in the MBS-model**. This figure shows a stick model which was built in Vicon Nexus^® ^allowing the measurement of the initial joint angles at the beginning of the recorded trials. The different segments of the stick model were generated by the recorded marker points used in the gait analysis and were linked via joints (hip joint, knee joint, ankle joint). Within Vicon Nexus^® ^an additional marker point in the middle of a connection line between the left and the right trochanter major is calculated which serves to determine the abduction/adduction angle. This figure also shows the initial position of the osseous segments of the hind limb in the preprocessor of the used MBS-system. The resulting joint angles between pelvis and femur, femur and tibia and between tibia and pad used in this MBS-model are shown in the table.

The sampling frequency for the kinematic data was 100 Hz, whereas the treadmill data were recorded with a frequency of 1000 Hz. In Vicon Nexus^® ^a stick model (Figure 2) was built, allowing the measurement of the initial joint angles at the beginning of the recorded trials. The different segments (pelvis, femur, tibia and pad) of the stick model were generated by the recorded marker points and were linked via joints (hip joint, knee joint, ankle joint). Within Vicon Nexus^® ^an additional marker point in the middle of a connection line between the left and the right trochanter major is calculated which serves to determine the abduction/adduction angle. The recorded trial data in Vicon Nexus^® ^were exported to an MS-Excel compatible file type (ASCII). The trial data included coordinate information in x-, y-, and z-direction for each of the recorded markers as well as the corresponding force data.

### Modeling of the bony structures

Computed tomographic (CT) data sets of a euthanized male mixed-breed dog with a body weight (BW) of 28 kg provided the basis for the development of the bony part of the canine MBS-model.

Pelvis, both femora, tibiae and pads were scanned after the removal of skin and subcutaneous tissue. The dog underwent a routine scan on a Philips Brilliance 64-Channel-CT scanner (Philips, Amsterdam, Netherlands). Spiral 1 mm CT sections (increment 0.5 mm, collimation 64 × 0.625, pitch 0.609) were obtained using the following parameters: kV 140, 250 mAS, 512 × 512 matrixes. The obtained DICOM-data were imported to *Analyze^® ^*(Mayo Clinic's Biomedical Imaging Resource (BIR) in Jacksonville, Florida) which is a commercially available software. After importing each single slice of the CT scan to *Analyze*^®^, the data had to be modified in order to build up a three-dimensional model from the two-dimensional DICOM-data. Based on the different Hounsfield units (HU) of the different anatomic structures on the CT scan, bony parts can be selected in *Analyze*^®^. A volume model of pelvis, both femora, tibiae and pads was generated, and exported to the multi-body simulation software BRG.LifeMOD as a neutral data format (igs-data). BRG.LifeMOD consists of a preprocessing unit, a solver and postprocessing unit. The preprocessing unit is used to define model-specific parameters (geometrical configuration of bones, joints, pad ground contact behavior, soft tissues) as well as characteristics concerning the movement of the model. In a second step the solver calculates the equations of motion using the Lagrangian approach. The postprocessing unit of BRG.LifeMOD is used for the visualization of the model in motion and for the analyses of e.g. forces, moments, joint angles and segment velocities and accelerations.

In the present study the initial position of the osseous segments of the hind limb (pelvis, femora, tibiae, pads) in the BRG.LifeMOD preprocessor were defined by the resulting joint angles between pelvis and femur, femur and tibia and between tibia and pad obtained from gait analyses, segment rotations were not taken into account (see table in Figure 2). After positioning the segments of the canine MBS-model, different masses were assigned to the different segments of the bones (Table 1). To determine bone mass, every bone was weighed (Sartorius, Göttingen, Germany) after detaching the soft tissues. The value of total bone density of the segments was calculated using the weight and volume of the bones (mean density *ρ*_1 _= 1.324 g/cm^3^). Because of the fact that the bony structure used in the model is based on only one dog, a sensitivity analysis was performed to determine how sensitive the model output is to the bony structure. Therefore the influence of three additional bone densities (*ρ*_2 _= 0.8 g/cm^3^, *ρ*_3 _= 1.8 g/cm^3^, *ρ*_4 _= 2.3 g/cm^3^) was investigated in comparison to *ρ*_1_.

**Table 1:** Weighed masses of used bone segments after detaching soft tissue

	pelvis	left femur	right femur	left tibia including fibula	right tibia including fibula	left foot	right foot
weight [g]	380	140	130	128	120	210	200

After implementation of the bone density, BRG.LifeMOD automatically calculated the mass, the centre of mass and the inertial properties for each bone segment. Once the segments of the model are established, joints are created between the segments. Joints are kinematic constraints which are used to connect two adjoining body segments. A joint consists of a tri-axis hinge and learns angulation patterns while the model is being driven by the motion capture data in an inverse dynamic simulation. Passive or active forces are acting on each of the three degrees of freedom of the particular joints. The joint angulation patterns are repeated and can afterwards serve as actuators for the forward dynamic simulation. In this MBS-model the pad ground interface was modeled as a single ellipsoid pad contact positioned in the plantar centre. The pad-ground contact was modeled using one spring-damper located in the center of the ellipsoid. The stiffness, damping and full damping depth values were assumed to be 11.4 N/mm, 0.18 Ns/mm and 0.17 mm, respectively. These parameters were modified from the study of Nazer et al. [20] to correspond to the gait analyses performed in this study.

### Integration of analogous muscle model

In addition to the implemented osseous structure of the canine hind limb-consisting of pelvis, both femora, tibiae and pads- the structure of soft tissue along with corresponding hip muscles was acquired by magnetic resonance imaging (MRI, Magnetom, Siemens, Munich, Germany). The generated muscle model was based on anatomical and morphological data of the right and left hind limb of four euthanized dogs having a mean body weight of 30 (± 2.78) kg. Using different image processing techniques (T1 and T2-weighted imaging) the coordinates of the muscles' origins and insertions on the bones (pelvis, femur, tibia) affecting the canine hind limb were extracted.

A standalone workstation (LEONARDO, SIEMENS; Germany) was used for acquiring and post-processing data from different imaging modalities (CT/MRI). In combination with *syngo*^® ^Software (Siemens, Germany), retrospective three-dimensional CT/MR image reconstructions were generated.

Twenty-two selected muscles of each hind limb were included in this study: the superficial gluteal, medial gluteal, deep gluteal, tensor fasciae latae (cranial and caudal part), rectus femoris, piriformis, gemelli, internal obturator, external obturator, quadratus femoris, gracilis, vastus lateralis and intermedius, vastus medialis, popliteus, biceps femoris, caudal crural abductor, semitendinosus, semimembranosus, gastrocnemius, tibialis cranialis medial, interosseous and extensor digitorum longus muscle (Figure 2).

For optimized presentation of the individual muscles the muscle bellies were separated, the inter-muscular space was filled with 3 mm thick foam and held in position by surgical fiber. Afterwards both hind limbs of each dog were scanned by MRI. Based on 3D-reconstructions of the muscles by the *syngo*^® ^software the origin and insertion of each muscle was defined and implemented in the existing MBS-model. In BRG.LifeMOD muscle elements have to be created in two phases based on the Hill-type approach for the mechanical modeling of muscles [20]. In the first "training" phase, muscle contractions are recorded in an inverse dynamic simulation. This is usually accomplished by using motion agents (virtual marker points similar to the kinematic markers in Vicon Nexus^®^; Figure 1) attached to the model. The motion agents are directed to move the model using time-displacement curve data for each axis of motion. In this current study the motion agents used to move the canine hind limb model are recorded during gait (see below).

In the second phase, after disabling the motion agents and updating the muscle specifications, the individual calculated contractile muscle characteristics are used in a forward dynamic simulation to achieve the desired contraction patterns.

In the multi-body simulation system BRG.LifeMOD the individual muscles are illustrated as straight lines (Figure 2). Due to a lack of literature about dynamic muscle forces during canine movements, published data concerning physiological cross-sectional areas (pCSA) and the related predicted maximal muscle force magnitudes (predicted by the minimization of the maximal muscle stress (MMMS) method) of the considered muscles of the canine hind limb were used for the implementation into the MBS-model [16,21,22]. Detached skin and subcutaneous tissues were weighed (9.38 kg) and implemented as a point mass linked to the pelvis segment in the MBS-model.

### Integration of kinematic data and validation of the MBS-model

The coordinate information for each marker recorded during the gait analyses was used to drive the virtual markers of the bony landmarks in the multi-body simulation system BRG.LifeMOD in the inverse dynamic simulation. The inverse dynamic simulation is used to capture the joint angle histories. During the simulation, the motion agents fulfill movements according to the prescribed data trajectories and each muscle is trained in order to accomplish the desired shortening/lengthening pattern to perform the given movement. The recorded patterns then serve as actuators to drive the motion in the forward dynamics simulation. A proportional derivative servo controller minimizes the error between the estimated contraction activities of the muscle and the actual muscle values obtained from the forward dynamics simulation.

**Figure 3:**
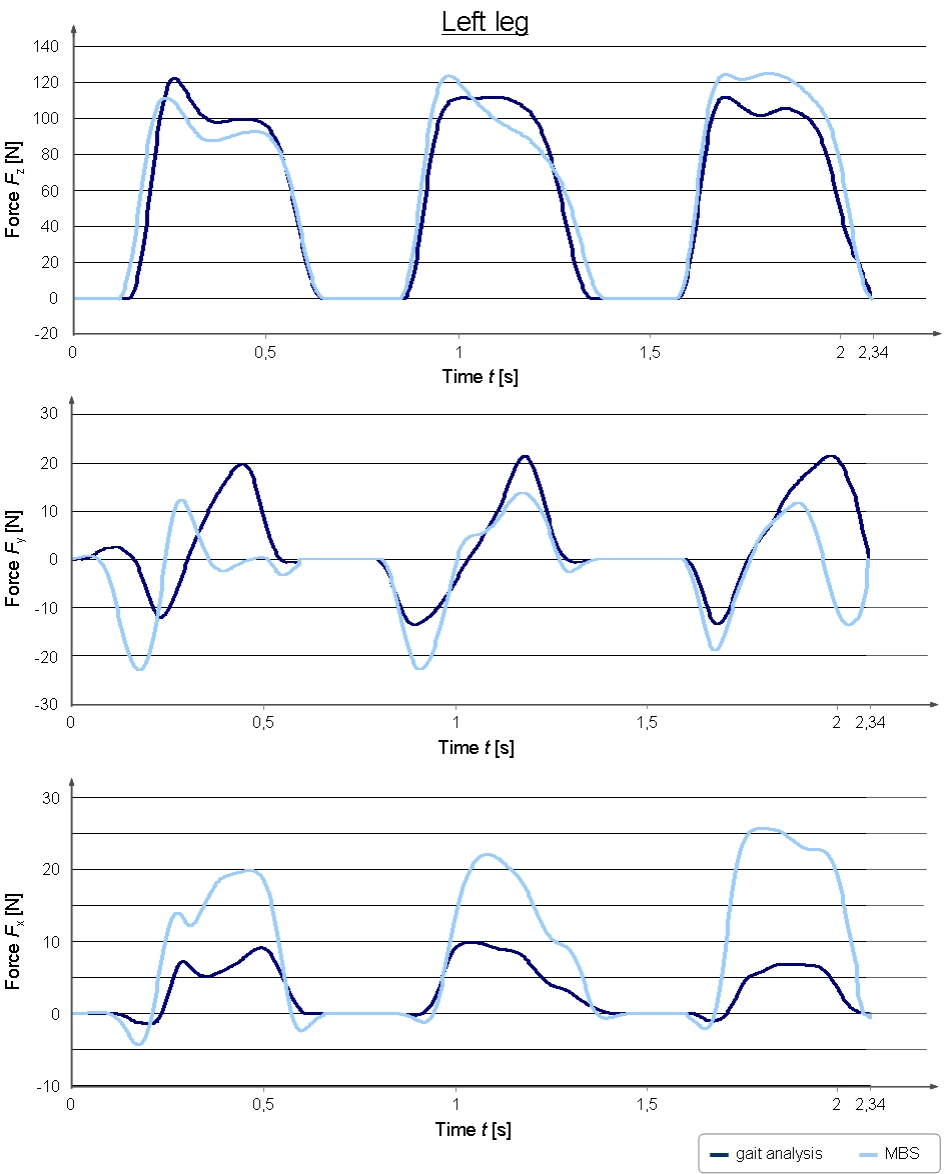
**Comparison between force plate measurements and the MBS-calculated ground reaction forces in x-, y- and z-direction for the left leg**. In this figure the measured and calculated ground reaction forces for the left leg in mediolateral (x-), craniocaudal (y-) and vertical (z-) direction are shown for three ground contacts of the respective pad.

**Figure 4:**
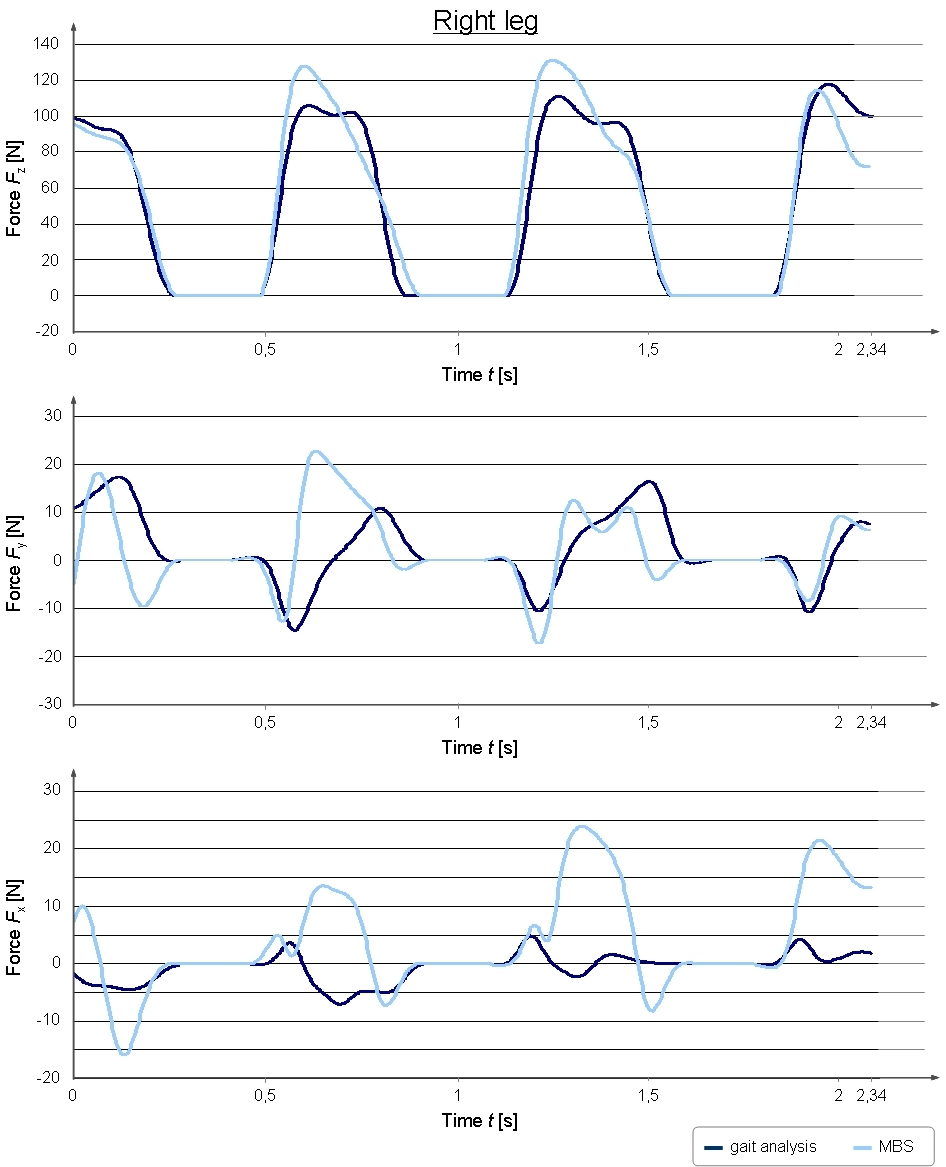
**Comparison between force plate measurements and the MBS-calculated ground reaction forces in x-, y- and z-direction for the right leg**. This figure shows the measured and calculated ground reaction forces for the right leg in mediolateral (x-), craniocaudal (y-) and vertical (z-) direction are shown for three ground contacts of the respective pad.

For the validation of the MBS-model treadmill data of the ground reaction forces of three valid ground contacts on each side were extracted (Figure 3, 4). The measured force plate data were filtered with a 4^th ^order zero phase-shift low-pass Butterworth filter with a frequency of 10 Hz. Mean peak values in x-, y- and z-direction from the treadmill measurements were compared descriptively with the values calculated in the MBS-model. Furthermore the correlation coefficient between treadmill measurements and MB simulations was investigated by means of the Spearman correlation test.

## Results

On the basis of both, CT- and MRI-data, a three-dimensional canine MBS-model of the hind limb was developed with an implemented analogous muscle model of 22 muscles (Figure 2). The orientation and initial position of the osseous segments of the hind limb in the MBS were taken from the gait analyses. As to be seen in Figure 2 the initial position of the hind limb in the MBS is exactly the same as in the stick model built up in Vicon Nexus^®^, the respective joint angles are displayed in the table of Figure 2. After integrating motion functions obtained from the kinematic analysis into the MBS-model, the canine gait pattern was represented in analogy to the recorded data (see additional file 1). Hence, the ground reaction forces in x-, y- and z-direction could be calculated and compared with the measured data.

The sensitivity analysis showed that different densities of the bony structure have an influence on the model output. For the different bony structures the mean peak vertical forces for the left limb differ by about 0.66%BW for *ρ*_2_, 6.31%BW for *ρ*_3 _and 8.37%BW for *ρ*_4 _in comparison to the bone density *ρ*_1 _used in the MBS model. For the right limb the mean peak vertical forces vary about 0.17%BW for *ρ*_2_, 6.41%BW for *ρ*_3 _and 8.21%BW for *ρ*_4 _in comparison to the bone density *ρ*_1 _(see additional file 2).

Figure 3 shows the simulated ground reaction forces for the left leg in comparison to the measured force values from the treadmill demonstrating that peak forces in the MBS-simulated condition were on the same level but slightly lower than the measurements from the treadmill. The measured mean peak vertical force value (41.98%BW) of the left hind limb in z-direction was found to be 1.75%BW lower than the value calculated by MBS (43.73%BW). In case of the occurring shear-forces (x-forces) during gait the mean peak values were found to be 3.12%BW (treadmill) and 8.17%BW (MBS) for the left hind limb. The force occurring in y-direction can be divided into a breaking and propulsion force [16]. The mean peak force values in y-direction were measured with 4.70%BW (breaking force, treadmill) and 7.54%BW (propulsion) in comparison to MBS values of 7.78%BW (breaking) and 4.60%BW (propulsion).

Figure 4 shows the simulated ground reaction forces for the right leg in comparison to the measured force values from the gait analyses. The peak vertical force (z-force) of the right hind limb was measured with a mean value of 40.63%BW (treadmill) and 45.28%BW (MBS) (Figure 4). The occurring shear-forces (x-forces) during gait were found to be 1.53%BW (treadmill) and 7.14%BW (MBS) for the right hind limb. The values for the right hind limb in y-direction were measured with 4.28%BW (breaking force, treadmill) and 4.27%BW (propulsion force) in comparison to the MBS values of 4.58%BW (breaking force) and 5.36%BW (propulsion force).

Both treadmill measurements of the left (r = 0.91) as well as of the right (r = 0.94) hind limb in z-direction showed a good correlation to the simulated data. A positive correlation between treadmill measurements and simulated x-forces could be demonstrated for the left (r = 0.84) and right (r = 0.31) hind limb. Measured and simulated y-forces were shown to have a positive correlation both, for the left (r = 0.49) and the right (r = 0.38) hind limb. The absolute values in y- and z-direction from the treadmill measurements and the MBS showed a very good analogy, whereas the force curve characteristics were similar only for the peak vertical force. The forces in x- and y- direction showed only in parts a good congruency between measured and simulated force curve characteristics.

## Discussion

The long-term success of total hip replacement depends on several factors. Among other causes the occurrence of wear debris is one of the main key issues in the development of particle induced osteolysis, which finally results in aseptic loosening of the prosthesis. Wear debris can develop over time influenced by the loading conditions in the hip joint. Therefore, the determination of loading conditions in the canine hip joint by means of a multi-body simulation model represents a decisive step towards an optimization of prosthesis components. In a first step, we have introduced the generation of a canine MBS-model of the hind limb for the simulation of ground reaction forces during different movements and its validation with force data obtained from an instrumented treadmill.

Various authors have evaluated and published findings on ground reaction forces, which occur in movements of the dog [16,17,23,24]. However, insufficient literature exists about forces and moments occurring in movements of dogs, although some valuable experimental data exist for instrumented canine hip joints [14]. To our knowledge up till now no three-dimensional MBS-model of the dog has been established, allowing the calculation of ground reaction forces as well as joint forces and moments during different movements. Simulations in general depend on the quality of the raw data, on which the model is generated from and admissible equations to simulate real situations.

Therefore the aim of the present study was to develop an MBS-model of the canine hind limb, which can be developed further to allow the determination of joint forces and moments in the different joints. In this study we calculated ground reaction forces during the canine gait cycle by means of the developed MBS-model and validated the results with measured gait analysis data.

For the kinematic analyses retroreflective markers were positioned on anatomical landmarks by means of double-sided adhesive tape. In general, recorded marker trajectories do not fully represent real movements of anatomical landmarks during gait due to unavoidable skin movements [24]. The problem of occurring marker vibrations can be avoided by small, light weighed markers strongly taped on the shaved skin [25]. In this context it has to be considered that the occurring error is growing with increasing gait velocity. The walking velocity of 1 m/s was chosen in this study to assure minor skin movements and hence a precise recording of the movement trajectories.

The movement simulation introduced here has shown that ground reaction can be calculated for the normal gait of the dog. Although the relationship between muscle activity and occurring joint forces is so far poorly understood, it is widely accepted that the ability of a muscle to generate force depends on its pCSA [15,18,21,22,26]. The concept of calculating muscle forces from pCSA represents an idealized method, because e.g. muscle-specific fiber types are not taken into consideration [22,23]. Since other more precise concepts are missing, we used the pCSA as an input parameter for the muscle activity during locomotion in the MBS. In order to allow the calculation of muscle forces (e.g. during isometric contractions) electromyographic (EMG) analyses have to be implemented into the MBS.

In the literature, data of segmental properties of the canine hind limb are available [27]. Unfortunately, these data cannot be implemented into the MBS introduced here. The automatically calculated inertial properties in BRG.LifeMOD depend on the bone density and volume of a segment. Therefore, an adjustment of the inertial properties according to the mentioned literature data would lead to a false automatic change of the original input parameter.

As a result, peak vertical forces of approximately 43.73%BW could be calculated in the MBS for the ground reaction forces for the left hind extremity (right hind limb: 45.28%BW) during the assumed sequence of walking. Furthermore, the measured forces ranged on the same level with peak vertical forces of 41.98%BW (left) and 40.63%BW (right). It could also be shown that different bone densities have an influence on the model output. As different dogs have varying bone densities, it is of great importance for a valid MBS to adjust the bone density parameter for every simulated patient. For the future utilization of the MBS-model for the calculation of hip joint loadings specific bone geometries like the contact area between femur and acetabulum or the coverage of femoral head by the acetabulum have to be taken into account.

Besides the good comparability between treadmill measurements and MBS for the vertical ground reaction forces our data are in good accordance with the literature values for dogs. Bockstahler et al. were able to demonstrate mean maximum vertical ground reaction forces in different dog breeds of 39.63%BW (left) and 39,1%BW (right) during walking on a treadmill [15]. In the same study it could be demonstrated that the differences in the peak vertical forces between the left and right hind limb show variances of up to 9.28% in clinically sound dogs [15]. This might also be an explanation for the differing values between left and right hind limb measured and simulated in this study.

As already demonstrated in other studies measured ground reaction forces in medio-lateral (x) direction differ a lot between each pad contact on a treadmill [16,28], which could be confirmed by the results presented here. Therefore, the informative value of this parameter is poor and negligible in gait analyses [16,28]. Nevertheless, we also tried to simulate ground reaction forces in x-direction. The demonstrated values for the measured and simulated breaking and propulsion forces in y-direction showed a good analogy in the absolute peak values, whereas the curve characteristics were not in complete alignment. The varying values between treadmill measurements and the MBS for the x- and y-force curve characteristics might be the result of the muscle specifications used in this study. In this context minimal deviations in the line of action of the acting muscles can lead to variations of the simulated force curve characteristics. One other reason for the variations between treadmill measurements and the MBS for the x- and y-force curve characteristics might be the modeling conditions of the pad-ground contact. In this context it has to be kept in mind that the contact specifications for the canine pad during gait are very difficult to determine, because the pad consists of five pad balls. In this MBS-model only a single point contact was defined between pad and ground. In further studies the model will be optimized with special regard to the pad-ground contact modeling.

## Conclusion

The results of this investigation show that peak ground reaction forces can be calculated by multi-body simulation using an inverse dynamics approach and that force curve characteristics for the z-direction are similar between simulated and measured conditions, whereas the curve characteristics in x- and y-direction are only partly acceptable. In summary, the developed MBS-model can serve as a basis for future investigations determining hip joint forces and moments as well as physiological muscle forces of the hind limb. In a further step, the estimated hip forces obtained from the MBS-model can be used for finite element analysis in order to evaluate the areas of high loadings in the inlay of canine hip prostheses [29]. These efforts enable an optimization of different prosthesis components based on simulation and hence help in reducing animal experiments.

## Competing interests

The authors declare that they have no competing interests.

## Authors' contributions

GH carried out the multi-body simulation, performed the analyses of the ground reaction forces and drafted the manuscript. Professor BAB is head of the IFUM. He was involved in the study design and supported the investigations. MS performed the multi-body simulation. PW carried out the gait analyses and drafted the manuscript. Professor IN is director of the Small Animal Clinic. He was involved in the study design and supported the investigations. All authors read and approved the final manuscript.

## Supplementary Material

Additional file 1 **MBS-model of the canine hind limbs and simulated gait cycle (.avi)**. This additional file shows the simulated canine gait cycle at a speed of 1 m/s in BRG.LifeMOD. At the beginning of the MBS the left pad is in contact to the ground while the right leg is in swing phase. Three pad-ground contacts on each side were simulated. During ground contact a red force vector can be seen, which represents magnitude and direction of the acting ground reaction force. Click here for file

Additional file 2 **Influence of different bone densities on the model output (sensitivity analysis)**. This figure shows that the mean peak vertical forces calculated in the MBS depends on the chosen bone density. The higher the bone density, the more increases the vertical ground reaction force calculated during the canine gait cycle. Click here for file
