# Supplementary figures and images for: Multi-body simulation of a canine hind limb: model development, experimental validation and calculation of ground reaction forces
Source: Biomed Eng Online. 2009 Nov 23;8:36. doi: 10.1186/1475-925X-8-36 (PMC2787502; doi:10.1186/1475-925X-8-36)

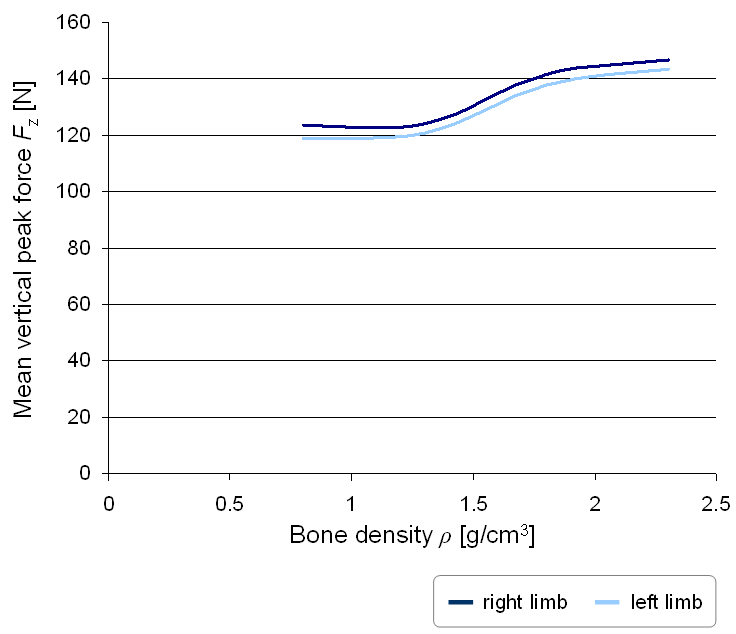

Supplement: Additional file 2 — Influence of different bone densities on the model output (sensitivity analysis). This figure shows that the mean peak vertical forces calculated in the MBS depends on the chosen bone density. The higher the bone density, the more increases the vertical ground reaction force calculated during the canine gait cycle. [file 1475-925X-8-36-S2.TIFF]
